# Supplementary material for: Molecular architecture and conservation of an immature human endogenous retrovirus
Source: Nat Commun. 2023 Aug 24;14:5149. doi: 10.1038/s41467-023-40786-w (PMC10449913; doi:10.1038/s41467-023-40786-w)
Supplement: Supplementary file 3 — Reporting Summary [file 41467_2023_40786_MOESM3_ESM.pdf]

nature portfolio

Corresponding author(s): Peijun Zhang
Last updated by author(s): Jul 25, 2023

Reporting Summary

Nature Portfolio wishes to improve the reproducibility of the work that we publish. This form provides structure for consistency and transparency in reporting. For further information on Nature Portfolio policies, see our Editorial Policies and the Editorial Policy Checklist.

Statistics

For all statistical analyses, confirm that the following items are present in the figure legend, table legend, main text, or Methods section.

n/a	Confirmed	
☐	☒	The exact sample size (*n*) for each experimental group/condition, given as a discrete number and unit of measurement
☒	☐	A statement on whether measurements were taken from distinct samples or whether the same sample was measured repeatedly
☒	☐	The statistical test(s) used AND whether they are one- or two-sided<br>*Only common tests should be described solely by name; describe more complex techniques in the Methods section.*
☒	☐	A description of all covariates tested
☒	☐	A description of any assumptions or corrections, such as tests of normality and adjustment for multiple comparisons
☐	☒	A full description of the statistical parameters including central tendency (e.g. means) or other basic estimates (e.g. regression coefficient) AND variation (e.g. standard deviation) or associated estimates of uncertainty (e.g. confidence intervals)
☒	☐	For null hypothesis testing, the test statistic (e.g. *F*, *t*, *r*) with confidence intervals, effect sizes, degrees of freedom and *P* value noted<br>*Give P values as exact values whenever suitable.*
☒	☐	For Bayesian analysis, information on the choice of priors and Markov chain Monte Carlo settings
☒	☐	For hierarchical and complex designs, identification of the appropriate level for tests and full reporting of outcomes
☒	☐	Estimates of effect sizes (e.g. Cohen's *d*, Pearson's *r*), indicating how they were calculated

Our web collection on statistics for biologists contains articles on many of the points above.

Software and code

Policy information about availability of computer code

Data collection	cryoEM data were collected using EPU (v2).
Data analysis	cryoEM data processing: IMOD (v4.11.7), EMAN2 (v2.91) and CSPT (v1.1).<br>Model building: RosettaCM (v3), Chimera (v1.17), ChimeraX (v1.4), VMD software version 1.9.4a57, Molprobity (v4.5.2).

For manuscripts utilizing custom algorithms or software that are central to the research but not yet described in published literature, software must be made available to editors and reviewers. We strongly encourage code deposition in a community repository (e.g. GitHub). See the Nature Portfolio guidelines for submitting code & software for further information.

Data

Policy information about availability of data

All manuscripts must include a data availability statement. This statement should provide the following information, where applicable:

- Accession codes, unique identifiers, or web links for publicly available datasets
- A description of any restrictions on data availability
- For clinical datasets or third party data, please ensure that the statement adheres to our policy

All data needed to evaluate the conclusions in the paper are present in the paper and/or the Supplementary information and source data are provided with this paper. The cryoET subtomogram averaging density maps and corresponding atomic models have been deposited in the EMDB and PDB, with accession codes EMD-16511 [https://www.ebi.ac.uk/emdb/EMD-16511] and PDB ID 8C9M [https://doi.org/10.2210/pdb8C9M/pdb], respectively.

Human research participants

Policy information about studies involving human research participants and Sex and Gender in Research.

Reporting on sex and gender	Not applicable
Population characteristics	Not applicable
Recruitment	Not applicable
Ethics oversight	Not applicable

Note that full information on the approval of the study protocol must also be provided in the manuscript.

Field-specific reporting

Please select the one below that is the best fit for your research. If you are not sure, read the appropriate sections before making your selection.

- [x] Life sciences
- [ ] Behavioural & social sciences
- [ ] Ecological, evolutionary & environmental sciences

For a reference copy of the document with all sections, see nature.com/documents/nr-reporting-summary-flat.pdf

Life sciences study design

All studies must disclose on these points even when the disclosure is negative.

Sample size	For cryo-EM SPA, sample sizes were those equired for the resolution. The details of datasets, including sample sizes, are listed in the Supplementary Table 1.
Data exclusions	During the cryoET STA study, the VLPs were manually picked and the particles were autopicked using uniformly distributed positions along the lattice. 2D and 3D classifications were performed to remove the junk particles in order to generate higher-resolution density maps.
Replication	Fro cryoET STA study, two randomly divided half datasets were processed independently and combined to give rise to the final structures. The resolution of the structure is assessed by comparing the two independent maps.<br>For western blot experiment, three replication experiments were performed and quantified. One of the representative images were presented in the figure. All attempts at replication were successful.
Randomization	For each dataset of CryoET STA study, the dataset were randomly divided into two half sets.
Blinding	The cryoET STA study including data collection and processing is not blinded, but were largely automated.

Reporting for specific materials, systems and methods

We require information from authors about some types of materials, experimental systems and methods used in many studies. Here, indicate whether each material, system or method listed is relevant to your study. If you are not sure if a list item applies to your research, read the appropriate section before selecting a response.

Materials & experimental systems

n/a	Involved in the study	
☐	☒	Antibodies
☐	☒	Eukaryotic cell lines
☒	☐	Palaeontology and archaeology
☒	☐	Animals and other organisms
☒	☐	Clinical data
☒	☐	Dual use research of concern

Methods

n/a	Involved in the study	
☒	☐	ChIP-seq
☒	☐	Flow cytometry
☒	☐	MRI-based neuroimaging

Antibodies

Antibodies used	Primary antibody against HERV-K Gag (HERM-1841-5, monoclonal, Amsbio) and secondary antibody anti-mouse, HRP conjugated (A0168 Sigma Aldrich) were used for Western blots.
Validation	The HERV-K Gag antibody has been approved for Western blots and ELISA by the manufacturer. It has been previously used to detect

HERV-K Gag:
George M, Schwecke T, Beimforde N, Hohn O, Chudak C, Zimmermann A, Kurth R, Naumann D, Bannert N. Identification of the protease cleavage sites in a reconstituted Gag polyprotein of an HERV-K(HML-2) element. Retrovirology. 2011 May 9;8:30. doi: 10.1186/1742-4690-8-30. PMID: 21554716; PMCID: PMC3114732.
Gonzalez-Hernandez MJ, Swanson MD, Contreras-Galindo R, Cookinham S, King SR, Noel RJ Jr, Kaplan MH, Markovitz DM. Expression of human endogenous retrovirus type K (HML-2) is activated by the Tat protein of HIV-1. J Virol. 2012 Aug;86(15):7790-805. doi: 10.1128/JVI.07215-11. Epub 2012 May 16. PMID: 22593154; PMCID: PMC3421662.
Jones RB, Garrison KE, Mujib S, Mihajlovic V, Aidarus N, Hunter DV, Martin E, John VM, Zhan W, Faruk NF, Gyenes G, Sheppard NC, Priumboom-Brees IM, Goodwin DA, Chen L, Rieger M, Muscat-King S, Loudon PT, Stanley C, Holditch SJ, Wong JC, Clayton K, Duan E, Song H, Xu Y, SenGupta D, Tandon R, Sacha JB, Brockman MA, Benko E, Kovacs C, Nixon DF, Ostrowski MA. HERV-K-specific T cells eliminate diverse HIV-1/2 and SIV primary isolates. J Clin Invest. 2012 Dec;122(12):4473-89. doi: 10.1172/JCI64560. Epub 2012 Nov 12. PMID: 23143309; PMCID: PMC3533554.

Eukaryotic cell lines

Policy information about cell lines and Sex and Gender in Research

Cell line source(s)	HEK293T initially bought from ECACC (12022001)
Authentication	Cell lines were not authenticated
Mycoplasma contamination	Cell lines were not tested for contamintation
Commonly misidentified lines (See ICLAC register)	Only HEK293T cells were used (CVCL_0045) which are not commonly misidentified.
